# Gastrointestinal Stromal Tumors (GIST): A Population-Based Study Using the SEER Database, including Management and Recent Advances in Targeted Therapy

**DOI:** 10.3390/cancers14153689

**Published:** 2022-07-28

**Authors:** Jaffar Khan, Asad Ullah, Abdul Waheed, Nabin Raj Karki, Nawaraj Adhikari, Lakshmi Vemavarapu, Sami Belakhlef, Samy Malik Bendjemil, Siamak Mehdizadeh Seraj, Feroze Sidhwa, Intisar Ghleilib, Shahin Foroutan, Andrew M. Blakely, Jaydira Del Rivero, Nagla Abdel Karim, Eric Vail, Saleh Heneidi, Hector Mesa

**Affiliations:** 1 Department of Pathology, Indiana University School of Medicine, Indianapolis, IN 46202, USA; khanja@iu.edu; 2 Department of Pathology, Vanderbilt University Medical Center, Nashville, TN 37232, USA; drasadkhankakar@gmail.com; 3 Department of Surgery, San Joaquin General Hospital, French Camp, CA 95231, USA; awaheed@sjgh.org (A.W.); smbendjemil@sjgh.org (S.M.B.); sseraj@sjgh.org (S.M.S.); fsidhwa@sjgh.org (F.S.); sforoutan@sjgh.org (S.F.); 4 Georgia Cancer Center, Department of Hematology Oncology, Medical College of Georgia, Augusta University, Augusta, GA 30912, USA; nkarki@augusta.edu; 5 Department of Medicine, Bon Secours Memorial Regional Medical Center, Mechanicsville, VA 23116, USA; nadhikari@soundphysicians.com; 6 Department of Pathology, Charlie Norwood Veterans Affairs Medical Center, Augusta, GA 30904, USA; lakshmi.vemavarapu@va.gov; 7 Department of Pathology, Medical College of Georgia, Augusta University, Augusta, GA 30912, USA; sbelakhlef@augusta.edu (S.B.); ighlelib@augusta.edu (I.G.); 8 National Cancer Institute (NCI), Bethesda, MD 20892, USA; andrew.blakely@nih.gov (A.M.B.); jaydira.delrivero@nih.gov (J.D.R.); 9 Inova Schar Cancer Institute, Department of Medicine, University of Virginia, Fairfax, VA 22031, USA; nagla.karim5@gmail.com; 10 Molecular Pathology Laboratory, Cedars-Sinai Medical Center, Los Angeles, CA 90048, USA; eric.vail@cshs.org (E.V.); saleh.heneidi@cshs.org (S.H.)

**Keywords:** SEER, GIST, metastatic GISTs, molecular, SDH, DOG1, spindle cell tumors

## Abstract

**Simple Summary:**

Gastrointestinal stromal tumors (GISTs) are the most common mesenchymal neoplasms of the gastrointestinal (GI) tract. All GISTs are potentially malignant and, in general, the risk is proportional to the tumor size. Due to their location within the muscularis propria, GISTs require an endoscopic ultrasound for intramural sampling, although jumbo forceps sampling can be diagnostic in ulcerated lesions. GISTs usually affect people aged between 60 and 70 years, with no gender predominance. Symptomatic tumors mostly present with GI bleeding, anemia, early satiety, and abdominal fullness. The main treatment of GISTs is complete surgical resection, ideally with the preservation of tumor integrity to prevent intra-abdominal dissemination due to rupture and spillage. Advanced-stage tumors require targeted therapy with tyrosine kinase inhibitors and multidisciplinary oncologic care.

**Abstract:**

Introduction: Gastrointestinal stromal tumors (GISTs) are the most common mesenchymal neoplasm of the gastrointestinal (GI) system. Most GISTs originate from the interstitial cells of Cajal (ICC), the pacemaker cell situated between the circular and longitudinal layers of the muscularis propria along the GI tract. In this population-based study using the SEER database, we sought to identify demographic, clinical, and pathologic factors that affect the prognosis and survival of patients with this neoplasm. Molecular genetic advances, current management guidelines, and advances in targeted therapy are discussed. Methods: Demographic and clinical data from GIST patients were retrieved from the SEER research plus database for the period 2000–2018. Statistical analysis was performed with IBM SPSS^®^ v20.2 software using the Chi-square test, paired t-test, multivariate analysis, and Kaplan–Meier functions. Results: A total of 10,833 patients with GIST were identified. Most patients were between 60–74 years of age: 40%, Caucasian: 68%, and the male to female ratio was 1.1:1. The most common primary tumor sites were stomach: 63%, small intestine: 30%, rectum: 3%, and esophagus: 0.7%. When reported, the grade of differentiation was well: 38%, moderately: 32%, undifferentiated: 19%, poorly: 12%. The size of most tumors ranged between 6–10 cm: 36% and they were treated by surgical intervention: 82% and/or chemotherapy/targeted therapy: 39%. The stage was localized: 66%, advanced: 19%, and regional: 15%. The 5-year survival was 74% (95% confidence interval (95% CI) = 72.6–74.7), and the 5-year cause-specific survival 82% (95% CI = 80.7–82.6). The 5-year cause-specific survival by treatment included surgery at 86% (95% CI = 85.4–87.3), chemotherapy/targeted therapy with or without surgery at 77% (95% CI = 75.7–78.9), and radiation at 75% (95% CI = 74.5–80). On multivariable analysis tumor size > 5 cm, poorly and undifferentiated grade, age > 60, and distant metastases at presentation were associated with worse overall survival. Conclusion: GISTs comprise 1–2% of malignancies of the GI tract, usually affect male Caucasians between the ages of 60 and 74 years, most tumors occur in the stomach and small intestine, and are usually >5 cm, but still localized, at the time of diagnosis. Most tumors receive multimodality surgical and chemotherapy/targeted therapy treatment, with a 5-year overall survival of 74% and cause-specific survival of 82%. GIST patients would benefit from enrollment in large clinical trials to establish better therapy guidelines for unresectable, treatment-refractory, and recurrent tumors.

## 1. Introduction

Gastrointestinal stromal tumors (GISTs) are the most common mesenchymal malignancy (sarcoma) of the gastrointestinal (GI) tract; however, they only comprise 1–2% of all GI malignancies. GISTs originate from pluripotential mesenchymal cells committed to become interstitial cells of Cajal (ICC), which are the pacemaker cells situated between the circular and longitudinal layers of the muscularis propria along the GI tract [1]. Most tumors affect the stomach and small intestine. The incidence of GIST is 10–15 cases per million worldwide, with ~5000 cases per year in the United States [2,3]. Small incidental lesions (tumorlets) are commonly identified during abdominal surgery, radiologic or endoscopic studies, and at autopsy [4]. Diagnosis relies on a combination of clinical, imaging, histopathology, immunohistochemistry, and molecular studies [5]. GISTs were formally recognized as a specific type of neoplasm in 2000, and their incidence has been increasing steadily due to improvements in diagnostic technologies [6]. Advanced-stage disease is reportedly present in 47% of cases, usually with metastases to the liver and peritoneum [7]. Symptomatic tumors manifest by a mass-like effect, leading to early satiety or obstruction, or symptoms associated with ulceration/tumor rupture, such as pain, bleeding, anemia, and perforation [8]. Most GISTs arise within the muscularis propria and appear as submucosal or mural masses in endoscopy or imaging [9]. Computed tomography (CT) is the gold standard for evaluating abdominal masses, since it provides information about the size, location, and presence or absence of regional and distant spread. The use of oral and intravenous contrast improves the evaluation of the tumor margins [10]. This study is one of the largest and most up-to-date database studies aimed at investigating the demographic, clinical, and pathological factors affecting the prognosis and survival of GIST patients, with special insights into emerging therapies.

## 2. Methods

The Surveillance, Epidemiology, and End Results (SEER) initiated by the National Cancer Institute in 1972 covers approximately 28% of the US population. SEER*Stat software version 8.4.0 (https://seer.cancer.gov/seerstat/, accessed 13 July 2022) was used to collect data from the database using international classification of disease version 3 (ICD-O-3), histological code 8936/3 and topographical codes C15.0, C15.1, C15.2, C15.3, C15.4, C15.5, C15.9, C16.0, C16.1, C16.2, C16.3, C16.4, C16.5, C16.6, C16.9, C17.0, C17.1, C17.2, C17.3, C17.9, C18.0, 18.1, C18.2, C18.3, C18.4, C18.5, C18.6, C18.7, C18.9, C19.9, C20.9, C21.0, C21.1, and C21.2.

Patients without microscopic confirmation of the diagnosis were excluded from the study. The data were exported to IBM’s Statistical Product and Service Solutions (SPSS©), version SPSS^®^ v20.2 (IBM Corp, Armonk, NY, USA). Demographic and clinical data, including age, race, sex, primary tumor site, histologic grading, tumor size, SEER-provided tumor pathological stage, treatment received (surgical, chemotherapy, and radiotherapy), overall survival, and survival by therapeutic modality were included. The SEER-provided chemotherapy treatment category refers predominantly to tyrosine kinase inhibitors targeting KIT and PDGFRA, since cytotoxic chemotherapy has been phased out as a systemic option. Endpoints included overall survival, mortality, and disease-specific survival. For categorical variables, the Chi-square test was used; for continuous variables, the paired t-test and analysis of variance (ANOVA) were used. To determine the independent factors affecting survival, multivariate analysis was used. *p <* 0.05 was considered statistically significant.

## 3. Results

Data from 10,833 patients were extracted from the SEER database for the period 2000–2018.

### 3.1. I- Demographic Characteristics

**Figure 1:**
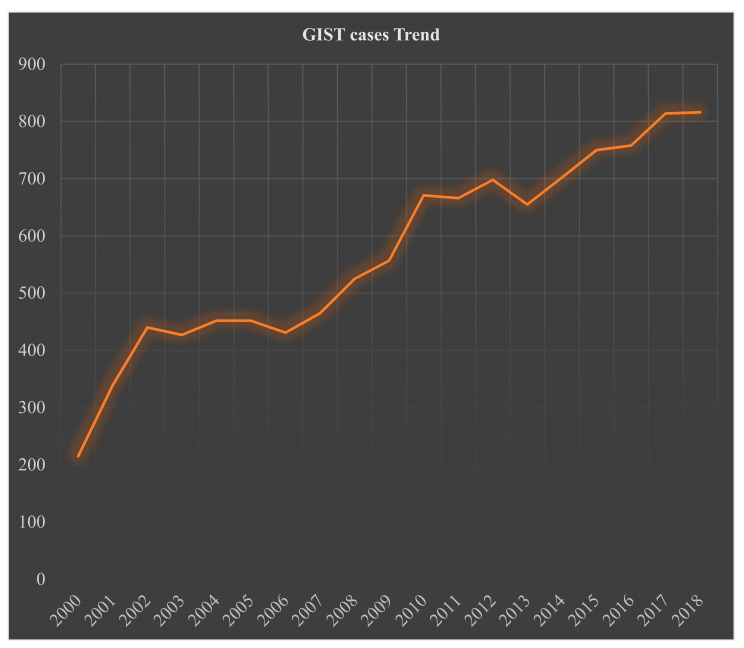
Trend analysis of 10,833 patients with gastrointestinal stromal tumor from the Surveillance, Epidemiology, and End Results (SEER) database 2000–2018.

By gender, 52% (*n* = 5633) of the patients were male and 48% (*n* = 5200, *p* < 0.035) female; the M:F ratio was 1.1:1. By age, most patients presented at ≥60 years (years): 63%, 45–59 years: 28%, 30–44 years: 8%, and only a few patients were <30 years: 2% (Table 1). Racial information was available for 99% of the patients: 68% were Caucasians, 19% African Americans, and 13% were classified as others (American Indians/AK Native, and Asian/Pacific Islanders), *p* < 0.001 (Table 1). The incidence of GIST has been increasing since a formal pathologic definition was established around the year 2000 (Figure 1).

### 3.2. I- Primary Site, Size, Grade, and SEER Stage

The most common tumor location was the stomach: 63%, followed by small intestine: 30% and rectum: 3%. The remaining sites comprised < 1% of the cases each. The tumor size was known in 74% of the cases: <2 cm: 7%, 2–5 cm: 29%, 6–10 cm: 36%, and >10 cm: 28%. The SEER tumor stage was known in 86% of the cases, reported as localized: 66%, regional involvement: 15%, or with distant metastases: 19%. The histologic grade was available in 43% of cases, reported as well-differentiated: 38%, moderately differentiated: 32%, undifferentiated: 19%, and poorly differentiated: 12% (Table 2). SEER does not provide data on the mitotic rate for GISTs.

### 3.3. I- Treatment

#### 3.3.1. Overall and Location Based

**Figure 2:**
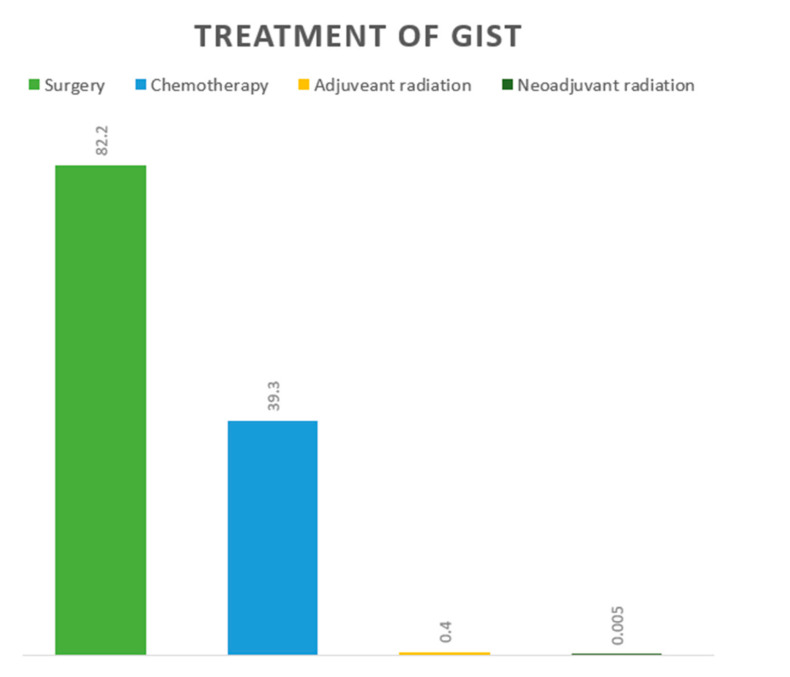
Treatment of 10,833 patients with gastrointestinal stromal tumors from the Surveillance, Epidemiology, and End Results (SEER) database 2000–2018.

Treatment data were available for most of the patients, and included surgery: 82%, chemotherapy with or without surgery: 39%, and radiation: 0.1% (Figure 2). Surgery was the most common treatment in almost all locations, followed by chemotherapy. Overall, the highest percentage of surgical resection was in appendicular GIST: 92%, followed by hepatic flexure: 78% and ascending colon: 78%. The highest percentage of chemotherapy with or without surgery was in GISTs located in the rectum: 43%, rectosigmoid: 42, and esophagus: 42%. Neoadjuvant radiation was only administered for GISTs located in the stomach and rectum (Table 3).

#### 3.3.2. Treatment According to Tumor Size, Stage, and Grade

Surgery was the primary treatment modality, irrespective of the tumor size and grade, followed by chemotherapy. Chemotherapy was the most common treatment modality in patients with distant spread (Table 4).

### 3.4. I- Overall and Cause-Specific Survival by Treatment

The overall cumulative survivals at 1, 3, and 5 years were 93% (95% CI = 92.2–93.3), 83% (95% CI = 82–83.7), and 74% (95% CI = 72.6–74.7), respectively. The disease-specific survivals at 1, 3, and 5 years were 95% (95% CI = 94.4–95.3), 88% (95% CI = 87.2–88.7), and 82% (95% CI = 80.7–82.6), respectively. Finally, the 5-year disease-specific survival with surgery was 86 % (95% CI = 85.4–87.3), and with chemotherapy ± surgery was 77% (95% CI 75.7–78.9) (Table 4). The 5-year cause-specific survival with adjuvant radiation was 67 % (95% CI = 47.5–80) (Table 5). Survival for radiation alone (0.1%) was not interpretable.

⮚ Five-year survival by location:

By location, the 5-year overall survival (OS) for GISTs was highest in the anus/anal canal: 88%, followed by rectum: 78%, transverse colon: 77%, small intestine: 75%, stomach: 73% and cecum: 73%. It was slightly lower for other locations, and lowest for ascending colon: 41% and appendix: 40% however these latter locations were too infrequent to draw any conclusions (Table 6).

⮚ Five-year disease-specific survival by treatment:

The 5-year disease-specific survival with surgical resection was highest when tumors were located in the transverse colon: 92%, followed by the rectum: 90%, anal region: 89%, stomach: 88%, and small intestine: 84%. When chemotherapy with or without surgery was used as the primary treatment modality, the highest 5-year disease-specific survival rates were observed for tumors located in the rectum: 89%, rectosigmoid: 86%, transverse colon: 84%, small intestine: 82%, and sigmoid colon: 80% (Table 7).

**Figure 3:**
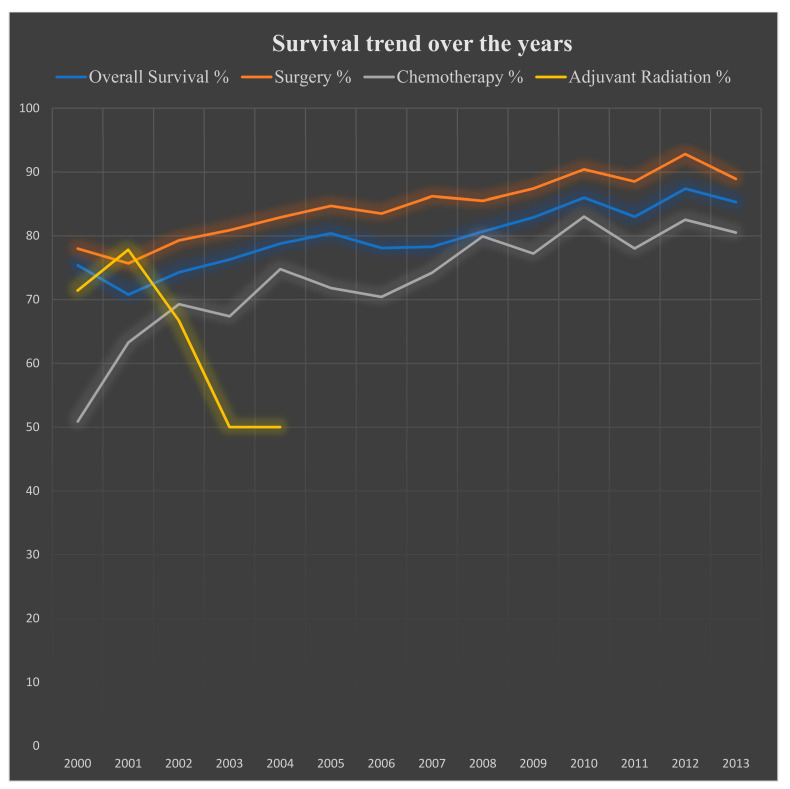
Survival trends over the years with different regimens used for 10,833 patients with gastrointestinal stromal tumors from the Surveillance, Epidemiology, and End Results (SEER) database 2000–2018. X-axis = Years; Y-axis = Percentage survival.

⮚ Survival trends for 5-year disease-specific survival by treatment:

The disease-specific survival greatly improved from 2000: 75% to 2013: 85% (*p* < 0.05). Surgery offered the best disease-specific survival in 2012: 93%; however. disease-specific survival has been increasing significantly with chemotherapy over the years, reaching its highest point in 2010: 83% (Table 8, Figure 3).

### 3.5. I- Univariate and Multivariable Analysis

According to multivariable analysis, the survival was most negatively affected by the tumor size being > 5 cm: hazard ratio (HR) = 7.30 (*p* < 0.001) and poorly differentiated and undifferentiated histology: HR = 5.35 (*p* < 0.001). Additional negative risk factors were location in the esophagus: HR = 3.62 (*p* < 0.001), age > 60 years: HR = 3.45 (*p* < 0.001), tumor with distant spread: HR = 3.17, and location in the ascending: HR = 2.53 and sigmoid colon: HR = 1.74 (*p* < 0.001) (Table 9).

## 4. Discussion

The histopathologic diagnosis of GIST relies on the expression of *c-KIT* and DOG1 (Discovered on GIST1) by immunohistochemistry [1]. Originally, all GISTs were believed to be derived from the interstitial cells of Cajal (ICC), the pacemaker cells of the GI tract; however, different molecular GIST subgroups have been identified more recently, leading to the realization that each subset may arise from other specialized stromal cells. Tumors originating from ICCs with KIT mutations are considered the “classic GIST” and were initially identified in 1998. They comprise 75% of GISTs, most commonly affect the stomach, and are responsive to imatinib therapy [11]. GIST with PDGFRA mutations make up the second-most-common group and comprise ~10% of all cases. PDGFRA Exon 12 and exon 18 mutations are more common in the duodenum and gastric antrum, respectively, and do not always respond to imatinib therapy [12]. The discovery that patients with germline PDGFRA mutations V561D and P653L develop multiple GISTs and inflammatory fibrous polyps in the stomach and small bowel led to the identification of a new PDGFRA+/CD34+/KIT+ interstitial stromal cell, the telocyte, which has been proposed as the cell of origin of PDGFRA-mutated GISTs with distinctive epithelioid histology and aggressive behavior [13,14,15].

GISTs without KIT or PDGFRA alterations are collectively dubbed “wild-type GISTs”, and make up the remaining 10–15% of all cases. This group is heterogeneous, not typically responsive to imatinib or other typical tyrosine kinase inhibitors (TKI), and includes SDH-deficient tumors (14%), tumors with mutations in NF1 (type 1 neurofibromatosis), BRAF V600E, and NTRK (0.5% each), and other rare mutations. SDH-deficient GISTs have loss-of-function alterations in one of the four SDH genes that encode for the subunits that constitute the SDH complex (mitochondrial succinate dehydrogenase complex), presenting in younger patients, and are usually only identified in the stomach. Loss-of-function alterations in SDHA/B/C/D are either due to sporadic epigenetic silencing (promoter hypermethylation) or germline alterations (Carney triad syndrome, Carney–Stratakis syndrome) at a ratio of 1:3 [16,17]. Animal models of BRAF V600E-driven GISTs develop tumors beyond the myenteric plexuses, which have been hypothesized to be derived from smooth muscle cells [18].

To our best knowledge, this study includes the largest cohort of GIST patients ever published. We found a 5-year overall cumulative survival of 74% (95% CI = 72.6–74.7), which is lower than that of the Dutch GIST Registry Study that reported a 5-year survival of 85% for adults aged 18–40 years and 76% for adults aged > 40 years [19]. Our study includes patients diagnosed between 2000 and 2018, while the Dutch study included patients diagnosed between 2009 and 2019 only. The improved survival in the Dutch cohort is explained by the more widespread use of TKIs over time, availability of universal health care in the Netherlands, different life expectancies of Dutch and American men and women, and earlier detection in the Dutch study: advanced disease 24% versus 34%.

In our study, age > 60 years and male gender were associated with worse overall survival, consistent with a previous SEER database study covering data from 1998 to 2011 [20]. In our study, non-gastric/non-small intestinal location (esophagus, and ascending and sigmoid colon) was associated with worse overall survival. Equivalent results were reported in another SEER database study with data from 1998–2011, [21] in which there were no differences in OS and DSS for small bowel, rectal, and gastric GISTs, but colonic and extra-visceral GISTs had significantly worse outcomes. A previous study from Iceland also showed lower metastatic rates in gastric than non-gastric GISTs [22]. A SEER database study from 1990–2009 showed that intestinal location had a higher risk of recurrence after surgery, independent of tumor size and mitotic rate, [23]; however, the interpretation of this data is difficult as GISTs were not defined as an entity until 2000. Together, all of these studies support better outcomes for gastric GISTs and worse outcomes for colonic GISTs. Tumors in very uncommon locations, such as the esophagus, rectum, and anus, and extra-gastrointestinal tumors are presumed to be at higher risk than gastric GISTs; however, reliable information is lacking due to their rarity. Like other sarcomas, large tumors >5 cm, distant metastases, and poor differentiation or undifferentiated, were associated with worse survival outcomes. Regarding treatment, surgery offered the best 5-year disease-specific survival, followed by systemic therapy with TKIs.

### 4.1. Targeted Treatments and Emerging Therapies

Complete surgical excision without lymph node dissection is the gold standard for localized tumors, similar to other visceral sarcomas [24,25,26]. Adjuvant imatinib for 3 years is indicated when a high risk of relapse exists; this benefit is unclear for wild-type GIST [27]. There is an ongoing phase-3 randomized trial comparing 3 vs. 5 years of adjuvant imatinib (Table 10. Newer TKIs are continuously emerging and being used for advanced, unresectable, relapsed, or metastatic GISTs based on the high prevalence of mutations leading to constitutively activated tyrosine kinase receptors (e.g., *c-KIT* 75%, *PDGFRA* 10–15%). Imatinib works best for *c-KIT* mutations in exon 11 (standard-dose imatinib), exon 9 (high-dose imatinib), and non-D842V *PDGFRA* mutations [28,29]. The *KIT* mutation A502_Y503dup comprises 90% of exon-9 mutations and is associated with locally aggressive tumors, spindle cell morphology, and decreased progression-free survival compared with exon 11 mutations, but better relapse-free survival after curative resection [30,31]. *KIT* exon-13 mutations are rare (1–2%) and most commonly occur as a secondary mutation after the initiation of therapy [32]. Esophageal and gastric *KIT* exon 13 mutant GISTs are rare, but tend to be larger and more aggressive on average. This has led them to be slightly overrepresented in clinical trials [32]. Exon-9, -13, and -14 *c-KIT* mutations can be treated with sunitinib. Exon-17 *c-KIT* mutations, which are usually the result of clonal evolution, may respond to ponatinib [28,29].

Avapritinib is recommended to treat symptomatic or progressive *PDGFRA* exon-18 D842V-mutated GISTs as an upfront therapy or whenever the mutation is discovered.

In practice, imatinib at a standard dose of 400 mg per day, sometimes followed by a trial of higher dose of 800 mg per day if disease progression occurs on the standard dose, sunitinib, regorafenib, and the recently approved ripretinib can be used for empiric treatment as first, second, third, and fourth lines for advanced GISTs, respectively [29,33,34]. These TKIs are not effective for *c-KIT* and *PDGFRA* wild-type GISTs (10–15% of total cases) [29,33,34]. Testing for alterations in *BRAF, NTRK, SDH*, and *NF1* should be pursued to detect targetable mutations treatable with BRAF inhibitors or BRAF–MEK inhibitor combinations and NTRK inhibitors, such as Larotrectinib [28]. There are 76 active studies registered on clinicaltrials.gov (accessed on 12 February 2022) enrolling patients with GISTs. GIST-driver mutations’ clinical characteristics and therapies are summarized in Table 11. A summary of active phase-3/4 treatment trials is provided in Table 10.

### 4.2. Limitations

The limitations of this study are mainly related to the quality and completeness of the information entered and retrievable from the database. The data regarding specific TKI treatment could not be extracted, data on genetic mutations and the mitotic index were not available, syndromic and sporadic cases could not be segregated from each other, and the type of surgical resection or margin status were not available, limiting the quality of the analysis.

## 5. Conclusions

Our study showed that tumor size > 5 cm and high histologic grade had the highest impact on survival. Additional negative risk factors were location other than the stomach and small bowel, age > 60 years, and distant spread. Female gender and primary surgery were associated with improved overall survival. Numerous existing and emerging targeted therapies are now available for high-risk or advanced disease. Optimal targeted therapy should be guided by genomic profiling, and this information should be collected in national databases to improve the existing treatment guidelines for unresectable or recurrent disease.

## Figures and Tables

**Table 1:** Demographics and trend data of 10,833 patients with gastrointestinal stromal tumors from the Surveillance, Epidemiology, and End Results (SEER) database 2000–2018.

Demographics		Frequency, Total = 10,833, (*n*, %)	*p*-Value
**Age in years**	0–14	19 (0.2)	<0.001
	15–29	153 (1.4)	
	30–44	857 (7.9)	
	45–59	2979 (27.5)	
	60–74	4280 (39.5)	
	>75	2545 (23.5)	
**Gender**	Male	5633 (52)	<0.035
	Female	5200 (48)	
**Race**	Unknown	84 (0.8)	<0.001
	Known	10,749 (99.2)	
	White	7340 (68.3)	
	African American	1992 (18.5)	
	Other (American Indian/Alaskan native, and Asian/Pacific Islander)	1417 (13.2)	
**Year 2000–2018**	**Trend**	**Count**	
% change	279.5	10,833	
Annual % change (APC)	5.3 *		*p* < 0.05

* The APC was calculated using the weighted least squares method.

**Table 2:** Tumor characteristics, grade, SEER stage, and location in 10,833 patients with gastrointestinal stromal tumors from the Surveillance, Epidemiology, and End Results (SEER) database 2000–2018.

Variable		Frequency Total = 10,833 (*n*, %)
**Primary Site**	Stomach	6868 (63.4)
	Small Intestine	3283 (30.3)
	Rectum	307 (2.8)
	Esophagus	71 (0.7)
	Large intestine (any site)	251 (2.3)
	Sigmoid colon	61 (0.6)
	Cecum	46 (0.4)
	Descending Colon	36 (0.3)
	Ascending Colon	36 (0.3)
	Large Intestine, NOS	32 (0.3)
	Transverse Colon	29 (0.3)
	Rectosigmoid Junction	19 (0.2)
	Hepatic Flexure	17 (0.2)
	Splenic Flexure	4 (0.03)
	Appendix	13 (0.1)
	Anus, anal canal, and anoderm	11 (0.1)
Stage *	Unknown	1523 (14)
	Known	9310 (86)
	Localized	6129 (65.8)
	Distant	1774 (19.1)
	Regional	1407 (15.1)
**Tumor Size**	Unknown	2833 (26.2)
	Known tumor size	8000 (73.8)
	<2cm	592 (7.4)
	2–5 cm	2306 (28.8)
	6–10 cm	2865 (35.8)
	>10 cm	2237 (28)
**Tumor Grade**	Unknown	6142 (56.7)
	Known	4691 (43.3)
	Well-differentiated	1768 (37.7)
	Moderately differentiated	1502 (32)
	Undifferentiated	867 (18.5)
	Poorly differentiated	554 (11.8)

* = The information related to the extent of the disease (EOD) was derived from the SEER summary-stage data collection system, which incorporates the EOD Primary Tumor, Regional Nodes, and Mets algorithm. EOD 0 = carcinoma in situ, 1 = organ-confined, 2 = regional extension, 3 = regional lymph nodes, 4 = direct extension, and regional lymph node involvement, 7 = distant extension, 8 = benign and borderline tumors, and 9 = extension and metastasis are unknown. (https://seer.cancer.gov/archive/manuals/2021/SPCSM_2021_MainDoc.pdf, accessed 13 July 2022).

**Table 3:** Treatment based on location for 10,833 patients with gastrointestinal stromal tumors from the Surveillance, Epidemiology, and End Results (SEER) database 2000–2018.

Known Location	Treatment When Known (*n*, %)			
	Surgery	Chemotherapy ± Surgery	Adjuvant Radiation	Neoadjuvant Radiation
Esophagus #	40 (56.3)	31 (42.3)	1 (1.4)	0
Stomach	5446 (68.3)	2502 (31.4)	16 (0.2)	5 (0.1)
Small Intestine	2946 (66.8)	1455 (33)	9 (0.2)	0
Cecum #	38 (73.1)	13 (25)	1 (1.9)	0
Appendix #	12 (92.3)	1 (7.7)	0	0
Ascending Colon #	31 (77.5)	9 (22.5)	0	0
Hepatic Flexure #	14 (77.8)	4 (22.2)	0	0
Transverse Colon #	24 (70.6)	10 (29.4)	0	0
Splenic Flexure #	3 (75)	1 (25)	0	0
Descending Colon #	34 (70.8)	13 (27.1)	1 (2.1)	0
Sigmoid Colon #	54 (75)	17 (26.6)	1 (1.4)	0
Large Intestine, NOS #	19 (65.5)	10 (35.5)	0	0
Rectosigmoid junction	14 (53.8)	11 (42.3)	1 (3.8)	0
Rectum #	216 (52.9)	174 (42.6)	17 (4.2)	1 (0.2)
Anus, Anal Canal, AD #	10 (66.7)	5 (33.3)	0	0

Abbreviations: NOS, Not otherwise specified; AD, Anoderm; #, Due to the small sample size, the data from these locations should be interpreted cautiously.

**Table 4:** Reported treatment based on the grade, size, and stage of 10,833 patients with gastrointestinal stromal tumor from the Surveillance, Epidemiology, and End Results (SEER) database 2000–2018.

**Tumor Size (*n*, %)**				
**Treatment**	**<2 cm**	**2–5 cm**	**6–10 cm**	**>10 cm**
Surgery	533 (90)	1796 (77.9)	1818 (63.4)	1283 (57.3)
Chemotherapy ± surgery	58 (9.8)	506 (21.9)	1042 (36.4)	947 (42.3)
Adjuvant radiation	1 (0.2)	4 (0.2)	4 (0.1)	6 (0.3)
Neoadjuvant radiation	0	0	1 (0.03)	1 (0.04)
**Tumor Grade (*n*, %)**				
**Treatment**	**Well** **differentiated**	**Moderately** **differentiated**	**Undifferentiated/** **anaplastic**	**Poorly** **Differentiated**
Surgery	1417 (80.1)	1062 (70.7)	523 (60.3)	338 (61)
Chemotherapy ± surgery	349 (19.7)	426 (28.4)	330 (38.1)	208 (37.5)
Adjuvant radiation	1 (0.05)	7 (0.5)	7 (0.7)	4 (0.7)
Neoadjuvant radiation	1 (0.05)	7 (0.5)	7 (0.7)	4 (0.7)
**Tumor Stage (*n*, %)**				
**Treatment**	**Localized**		**Distant**	**Regional**
Surgery	4895 (73.6)		1005 (44.9)	1245 (63.8)
Chemotherapy ± surgery	1735 (26.1)		1222 (54.6)	690 (35.4)
Adjuvant radiation	19 (0.3)		10 (0.4)	12 (0.6)
Neoadjuvant radiation	1 (0.01)		1 (0.04)	4 (0.2)

**Table 5:** Overall cumulative and disease-specific survival data of 10,833 patients with gastrointestinal stromal tumors from the Surveillance, Epidemiology, and End Results (SEER) database 2000–2018.

**Overall Cumulative Survival (%, 95% Confidence Interval)**	
1-year	92.7 (92.2–93.3)
2-year	88.1 (87.4–88.8)
3-year	82.9 (82–83.7)
4-year	78.3 (77.3–79.2)
5-year	73.7 (72.6–74.7)
**Disease-specific survival**	
1-year	94.9 (94.4–95.3)
2-year	91.7 (91–92.2)
3-year	87.9 (87.2–88.7)
4-year	85 (84.1–85.8)
5-year	81.6 (80.7–82.6)
**5-year disease-specific survival by treatment**	
Surgery	86.4 (85.4–87.3)
Chemotherapy	77.4 (75.7–78.9)
Adjuvant Radiation	66.5 (47.5–80)

**Table 6:** Observed survival data by location for 10,833 patients with gastrointestinal stromal tumors from the Surveillance, Epidemiology, and End Results (SEER) database 2000–2018.

Location	Observed Survival, % (95 Confidence Interval)				
	1-Year	2-Year	3-Year	4-Year	5-Year
Esophagus #	79.4 (65.1–88.3)	79.4 (65.1–88.3)	74.6 (59.6–84.8)	74.6 (59.6–84.8)	63 (46.4–75.8)
Stomach	92.5 (91.7–93.1)	87.7 (86.8–88.6)	82.3 (81.2–83.4)	77.7 (76.4–78.8)	73.2 (71.9–74.5)
Small Intestine	94.2 (93.2–95)	89.8 (88.6–90.9)	84.6 (83.1–86)	79.8 (78.1–81.4)	75.2 (73.3–76.9)
Cecum #	83.8 (65.2–92.9)	76.8 (57.3–88.2)	72.8 (52.6–85.4)	72.8 (52.6–85.4)	72.8 (52.6–85.4)
Appendix #	+	+	80 (20.4–96.9)	60 (12.6–88.2))	40 (5.2–75.3))
Ascending Colon #	69 (48.8–82.5)	56.4 (35.9–72.7)	51.7 (31.2–68.9)	51.7 (31.2–68.9)	41.4 (21.6–60.2)
Hepatic Flexure #	84.4 (50.4–95.9)	67.5 (34.6–86.5)	67.5 (34.6–86.5)	67.5 (34.6–86.5)	54 (20.3–78.9)
Transverse Colon #	89.5 (641–97.3)	89.5 (641–97.3)	76.7 (48.8–90.6)	76.7 (48.8–90.6)	76.7 (48.8–90.6)
Splenic Flexure #	+	66.7 (5.4–94.5)	66.7 (5.4–94.5)	66.7 (5.4–94.5)	66.7 (5.4–94.5)
Descending Colon #	86.3 (67.5–94.6)	78.6 (58.2–89.8)	74.2 (53.1–86.9)	69.6 (47.8–83.7)	64.6 (42.3–80.1)
Sigmoid Colon #	91 (77.8–96.5)	79 (63.5–88.5)	73.8 (57.6–84.6)	70.9 (54.2–82.4)	67.9 (51–80.1)
Large Intestine, NOS #	78.9 (58.9–89.9)	78.9 (58.9–89.9)	71 (50.2–84.4)	62.7 (41.4–78)	50.1 (29.7–67.5)
Rectosigmoid Junction #	78.6 (47.2–92.5)	78.6 (47.2–92.5)	78.6 (47.2–92.5)	78.6 (47.2–92.5)	69.8 (37.8–87.6)
Rectum #	93.3 (89.4–95.8)	89.8 (85.3–93)	87.5 (82.6–91.1)	84.5 (79.2–88.6)	78.7 (72.6–83.6)
Anus, Anal Canal, AD #	+	88.9 (43.3–98.4) ^&^	88.9 (43.3–98.4) ^&^	88.9 (43.3–98.4) ^&^	88.9 (43.3–98.4) ^&^

Abbreviations: NOS, Not otherwise specified; AD, Anoderm; +, statistics could not be calculated; &, width of the confidence interval is more than that if normal approximation is applied; # = Due to the small sample size, the survival data from these locations should be interpreted cautiously.

**Table 7:** Five-year disease-specific survival data by treatment modality and location for 10,833 patients with gastrointestinal stromal tumors from the Surveillance, Epidemiology, and End Results (SEER) database 2000–2018.

Location	5-Year Disease-Specific Survival, (%, 95 Confidence Interval)		
	Surgery	Chemotherapy ± Surgery	Adjuvant Radiation
Esophagus #	79.6 (57.4–91.1)	62.1 (33–81.5)	+
Stomach	88.4 (87.2–89.5)	74.4 (72.1–76.5)	70.7 (33.7–89.5)
Small Intestine	83.7 (81.9–85.3)	81.7 (79–84)	57.1 (17.2–83.7)
Cecum #	79.5 (57.4–90.9)	64.8 (25.3–87.2)	+
Appendix #	+	+	+
Ascending Colon #	43.9 (21.7–64.2)	33.3 (5.3–66.4)	+
Hepatic Flexure #	48.5 (14.4–76.3)	+	+
Transverse Colon #	92.3 (56.6–98.9) ^&^	83.3 (27.3–97.5)	+
Splenic Flexure #	+	+	+
Descending Colon #	73.6 (49.5–87.5)	51.1 (13.8–79.7)	+
Sigmoid Colon #	77.1 (59.1–87.9)	80 (40.9–94.6)	+
Large Intestine, NOS #	67.6 (38.6–85.1)	60 (79–85.5)	+
Rectosigmoid Junction #	78.8 (38.1–94.3) ^&^	85.7 (33.4–97.9)	+
Rectum #	90.3 (84.3–94)	88.5 (80.5–93.4)	66.6 (33.1–86.1)
Anus, Anal Canal, AD #	88.9 (43.3–98.4) ^&^	+	+

Abbreviations: NOS, Not otherwise specified; AD, Anoderm; +, Statistics could not be calculated; &, Width of the confidence interval is more than that if normal approximation is applied; #, Due to the small sample size, the survival data from these locations should be interpreted cautiously.

**Table 8:** Five-year disease-specific survival trend data by treatment over the years for 10,833 patients with gastrointestinal stromal tumor from the Surveillance, Epidemiology, and End Results (SEER) database 2000–2018.

	5-Year Disease-Specific Survival, % (Confidence Interval)			
Year	Overall Disease-Specific Survival	Surgery	Chemotherapy ± Surgery	Adjuvant Radiation
2000	75.4 (68.1–81.2)	78 (70.5–83.7)	50.9 (28.9–69.2)	71.4 (25.8–92)
2001	70.8 (64.9–75.8)	75.7 (69.7–80.7)	63.3 (50.1–73.9)	77.8 (36.5–93.9)
2002	74.3 (69.3–78.6)	79.3 (74.3–83.5)	69.3 (58.7–77.7)	66.7 (5.4–94.5)
2003	76.3 (71.3–80.6)	80.9 (75.8–85)	67.4 (57.4–75.5)	50 (11.1–80.4)
2004	78.8 (74.1–82.7)	82.9 (78.1–86.7)	74.8 (64.7–82.3)	50 (0.6–91)
2005	80.4 (75.8–84.2)	84.7 (80–88.3)	71.8 (62.1–79.3)	NA
2006	78.1 (73.2–82.2)	83.5 (78.5–87.4)	70.4 (60.6–78.1)	NA
2007	78.3 (73.7–82.2)	86.2 (81.8–89.7)	74.2 (67–80.2)	NA
2008	80.7 (76.5–84.3)	85.5 (81.3–88.8)	79.9 (73.7–84.8)	NA
2009	82.9 (79–86.1)	87.4 (83.5–90.4)	77.2 (70.6–82.4)	NA
2010	86 (82.6–90.8)	90.4 (87.2–92.8)	83 (77.7–87.1)	NA
2011	83 (79.4–86)	88.5 (85–91.3)	78 (72–82.8)	NA
2012	87.4 (84.2–90)	92.8 (89.9–94.9)	82.5 (76.9–86.9)	NA
2013	85.3 (81.8–88.1)	88.9 (85.5–91.5)	80.5 (74.7–85.1)	NA
2014–18	NA	NA	NA	NA

Abbreviations: NA, Not available.

**Table 9:** Univariate and multivariate analysis of factors affecting mortality among 10,833 patients with gastrointestinal stromal tumors from the Surveillance, Epidemiology, and End Results (SEER) database 2000–2018.

Variables		Univariate	Multivariate Analysis	
		*p*-Value	Hazard Ratio (95% Confidence Interval)	*p*-Value
Age > 60 Years		<0.001	3.45 (1.81–6.61)	<0.001
Gender	Male	0.767		
	Female			
Location	Esophagus	0.004	1.82 (1.25–2.72)	0.004
	Stomach	0.129		
	Small Intestine	0.847		
	Cecum	0.052		
	Appendix	0.316		
	Ascending Colon	0.003	2.53 (2.35–3.21)	0.003
	Hepatic Flexure	0.651		
	Transverse Colon	0.542		
	Splenic Flexure	0.212		
	Descending Colon	0.231		
	Sigmoid Colon	0.004	1.74 (1.28–2.34)	0.004
	Large intestine, NOS	0.855		
	Rectosigmoid Junction	0.658		
	Rectum	0.645		
	Anus, Anal Region, Anoderm	0.669		
Size	<2 cm in size	0.997		
	2–5 cm in size	0.757		
	>5 cm in size	0.01	7.30 (1.11–47.87)	0.003
Stage	Localized	0.733		
	Regional	0.512		
	Distant	0.003	3.17 (1.26–7.24)	0.001
Grade	Well-Differentiated	0.679		
	Moderately Differentiated	0.13		
	Poorly Differentiated	0.003	5.35 (2.92–12.1)	<0.001
	Undifferentiated	0.003	5.35 (2.92–12.1)	<0.001

**Table 10:** Selected ongoing phase-3 treatment trials enrolling patients with gastrointestinal stromal tumors (GISTs): (Source: Clinicaltrials.gov, accessed 12 February 2022).

Trial Number (Name)	Study Title	Study Type	Study Arms	Primary Outcome	Status (on 12 Feb 2022)
NCT04409223	Efficacy and safety of famitinib vs. sunitinib for advanced GIST after failure of imatinib	Phase 3, randomized	Famitinib vs. sunitinib	PFS	Recruiting
NCT00756509	Treatment of patients with metastatic or unresectable GIST in first line with nilotinib	Phase 4, single-arm	Nilotinib	Rates of stable SD, PR, and CR	Active, not recruiting
NCT05208047 (Peak)	A phase 3 randomized trial of CGT9486 + sunitinib vs. sunitinib in subjects with GIST	Phase 1a and phase 3, randomized	CGT9486 + sunitinib vs. sunitinib	Pharmacokinetics (Cmax, AUC, Tmax, T1/2, CLss/F) and PFS	Recruiting
NCT03673501 (Intrigue)	A study of DCC-2618 vs sunitinib in advanced GIST after treatment with imatinib	Phase 3, randomized	DCC-1618 (ripretinib) vs. sunitinib	PFS	Active, not recruiting
NCT03353753 (INVICTUS) *	Phase 3 study of DCC-2618 vs. placebo in advanced GIST treated with prior anticancer therapies	Phase 3, randomized	DCC-1618 (ripretinib) vs. placebo	PFS	Active, not recruiting
NCT04825574	Study for patients previously treated in avapritinib clinical trials	Phase 4	Avapritinib	Safety	Active, not recruiting
NCT02260505 (ImadGist)	Efficacy of imatinib maintenance or interruption after 3 years of adjuvant treatment in patients with GIST	Phase 3, randomized	Maintenance imatinib	DFS	Recruiting
NCT02847429	Randomized trial of crenolanib in subjects with D842V mutated GIST	Phase 3, randomized	Crenolanib vs. placebo	PFS	Active, not recruiting
NCT02413736	3 vs. 5 years of adjuvant Imatinib in patients with operable GIST with a high risk for recurrence: A randomized phase III study	Phase 3, randomized	Experimental: Imatinib at 400 mg/day for 24 months	RFS	Recruiting

Abbreviations; PFS, progression-free survival; SD, stable disease; PR, partial response; CR, complete response; Cmax, maximum plasma concentration; AUC, area under the plasma concentration–time curve, Tmax, time to maximum observed plasma concentration; T1/2, time to plasma concentration terminal half-life; CLss/F, apparent total body clearance at steady state; DFS, disease-free survival, RFS, recurrence-free survival. * Results published already.

**Table 11:** GIST mutations’ clinical course and management. **High Risk ** is in bold and underlined.

Gene	Alteration	Clinical Features	Recommended Targeted Therapy (Potential Superior Therapy)
KIT	Exon 11 **W557_558del**	Classic GISTs (V559 and V560) W557 more aggressive in the stomach Half W557 non-gastric, avg. 8 cm	Imatinib standard dose
	Exon 9 **A502_Y503dup**	Locally aggressive, spindle cell Non-gastric, younger, >5 cm	Potential adjuvant therapy Imatinib high dose Sunitinib
	Exons 13, 14, and 17	Secondary mutations resistant to imatinib/sunitinib	Sunitinib and ponatinib
PDGFR	Exon 18 D842V	PDGFRA alteration, mostly gastric Favorable outcomes Resistance to imatinib and sunitinib	Avapritinib No adjuvant therapy recommended Neoadjuvant avapritinib may be considered
	Exons 13–15 Codons 596–719	Resistance to avapritinib	Imatinib standard dose (Trametinib)
	Exon 12 Codons 555–589	Primary non-gastric GISTs, rare	Imatinib standard dose
SDHA-D	Hypermethylation, truncation, frameshift, Splice site alterations	Carney triad syndrome (often SDHC) Multifocal gastric GISTs, pulmonary chondroma paraganglioma Younger with female > male Carney–Stratakis syndrome Germline, gastric GISTs, and paraganglioma Paternal inheritance (SDHD) Potential lymph node metastasis	Avoid TKIs Personalized treatment
BRAF	V600E	Resistant to standard GIST guideline TKIs	Off-label indication of BRAF and BRAF–MEK inhibitors
NF1	Truncation Frameshift	Germline Most GIST in small bowel	Avoid adjuvant therapy Personalized treatment
NTRK1,2,3	Fusions	Resistant to standard GIST guideline TKIs	NTRK inhibitors (Larotrectinib and entrectinib)

## Data Availability

All data are publicly available. The data of this manuscript were presented at the Society of American Gastrointestinal and Endoscopic Surgeons (SAGES), Las Vegas, Nevada, 31 August–3 September 2021.
